# A Novel q-Rung Dual Hesitant Fuzzy Multi-Attribute Decision-Making Method Based on Entropy Weights

**DOI:** 10.3390/e23101322

**Published:** 2021-10-11

**Authors:** Yaqing Kou, Xue Feng, Jun Wang

**Affiliations:** 1 School of Economics and Management, Beijing Jiaotong University, Beijing 100044, China; 20120605@bjtu.edu.cn (Y.K.); 19120604@bjtu.edu.cn (X.F.); 2 School of Economics and Management, Beijing University of Chemical Technology, Beijing 100029, China

**Keywords:** multi-attribute decision-making, q-rung dual hesitant fuzzy sets, power average, Hamy mean

## Abstract

In this paper, a new multiple attribute decision-making (MADM) method under q-rung dual hesitant fuzzy environment from the perspective of aggregation operators is proposed. First, some aggregation operators are proposed for fusing q-rung dual hesitant fuzzy sets (q-RDHFSs). Afterwards, we present properties and some desirable special cases of the new operators. Second, a new entropy measure for q-RDHFSs is developed, which defines a method to calculate the weight information of aggregated q-rung dual hesitant fuzzy elements. Third, a novel MADM method is introduced to deal with decision-making problems under q-RDHFSs environment, wherein weight information is completely unknown. Finally, we present numerical example to show the effectiveness and performance of the new method. Additionally, comparative analysis is conducted to prove the superiorities of our new MADM method. This study mainly contributes to a novel method, which can help decision makes select optimal alternatives when dealing with practical MADM problems.

## 1. Introduction

Multi-attribute decision-making (MADM) indicates a series of decision-making problems that we often encounter in our daily life [1,2,3,4,5]. MADM theories and methods have received great interests and quite a few significant achievements have been published [6,7,8,9,10]. At the same time, these theories have also been applied in many fields to solve practical problems [11,12,13,14], such as the prevention of soil erosion [11] and factory location selection [12]. There are many kinds of methodologies to deal with MADM issues and aggregation operators are impressive tools, as they integrate individual attribute values into single ones. Decision makers (DMs) can easily and conveniently get the rank of feasible alternatives according to their overall evaluation values by using aggregation operators. However, it is not easy to aggregate attribute values in actual MADM problems, as there exists complicated and daedal interrelationship among attributes. Hence, in the process of calculating the overall evaluation values of alternatives, the interrelationship among the attribute values ought to take into account.

Based on these facts, more and more researchers and scholars have started to investigate to fuse attribute values from the perspective of Bonferroni mean (BM) [15] and HEronian mean (HEM) [16]. The attractive and prominent characteristic of BM and HM is their ability of considering the interrelationship that is subsistent among attribute values. It is worthy pointing out that BM and HM were originated for crisp numbers and in order to adopt them to different complicated and fuzzy decision environment, BM and HM has been extended to accommodate fuzzy decision-making information.

On the other side, the q-rung dual hesitant fuzzy sets (q-RDHFSs) proposed by Xu and her colleagues [17] is an effective tool to depict assessment information of DMs, and they absorb advantages of both q-rung orthopair fuzzy sets (q-ROFSs) [18] and dual hesitant fuzzy sets (DHFSs) [19]. In [17], Xu and her colleagues investigated aggregation operators of q-RDHFEs and applied them in decision-making problems. Afterwards, some extensions of q-RDHFSs have also been put forward and deeply studied, which also illustrate the uniqueness and superiorities of q-RDHFEs in dealing with fuzzy and uncertain information [20,21,22,23,24]. Nonetheless, we must point out that existing MADM method based on q-RDHFSs still has some shortcomings. First, existing method only considers the interrelationship among attribute values, whereas fails to further consider how to effectively deal with DMs’ unreasonable or extreme assessment values. In other words, when DMs provide absurd the decision-making results, Xu et al.’s [17] method produces unreasonable results. Besides, the method proposed by Xu et al.’s [17] only considers situations where the weight information of attributes is completely known. However, in most practical MADM problems, the weight vector of attributes is unknown. Hence, the novel MADM method that aims to solve MADM problems under q-RDHFSs with unknown weight information is highly necessary.

The main novelties and motivations of our paper can be summarized as follows. (1) Novel aggregation operators for fusing q-rung dual hesitant fuzzy information are proposed. Considering the good performance of the power Hamy mean (PHM) in aggregating fuzzy information [24,25,26], we extend it into q-RDHFSs and introduce novel aggregation operators for q-RDHFSs. These operators noy only consider the interrelationship between attributes but also effectively handle DMs’ unreasonable or extreme evaluation values. (2) A new method to determine the weight vector of attributes is proposed. In most practical MADM problems, weight information of attributes is usually unknown. In addition, entropy is widely used to determine attributes’ weights. Hence, this study presents entropy measure for q-RDHFSs and based on which, a method to calculate weights in MADM under q-RDHFSs environment is introduced. (3) We give a new MADM method to deal with decision-making problems under q-RDHFSs with unknown weight information. Meanwhile, in order to prove the practical value of this method, we also conduct numerical analysis.

The rest of this paper is organized as follows. Section 2 reviews related literature. Section 3 recalls basic concepts that will be used in later sections. Section 4 studies novel aggregation operators for q-RDHFEs and investigates their properties. Section 5 investigates entropy of q-RDHFEs and shows the process of determining weight information. Section 6 introduces a new MADM method with q-RDHFEs. Section 7 demonstrates the actual performance of the new method through numerical examples. Summarization and future research directions are presented in Section 8.

## 2. Literature Review

As the complexity of decision-making problems increases, it is very difficult to use clear values to describe attribute values. Therefore, more and more scholars are concerned about how to deal with this uncertain phenomenon. Zadeh [27] constructed the concept of fuzzy set (FS), which only has a membership degree (MD), thereby it is impossible to describe the imprecision. Atanassov [28] presented an intuitionistic fuzzy set (IFS) to deal with the fuzziness and uncertainty in 1986. To overcome the limitation of IFS, Yager [29] introduced concept of the Pythagorean fuzzy set (PFS), which can enable the cases of the sum of the MD and non-membership degrees (NMD) is larger than one. In 2017, Yager [18] proposed the concept of q-ROFS to cope with situations wherein the square sum of MD and NMD exceeds one. In real decision-making problems, the DMs may hesitate in a set of values when determines the attribute value, Therefore Torra [30] presented the concept of hesitating fuzzy set (HFS). Due to the limitation of HFS, Zhu et al. [19] proposed the concept of dual hesitant fuzzy set (DHFS), which can both represent the MD and NMD. Xu et al. [17] expanded the concepts of q-ROFSs and DHFSs and presented q-RDHFSs, to describe uncertain phenomena.

With the development of fuzzy sets, their aggregation operators are discussed widely. The main works and contributions of scholars are to extend BM and HM to accommodate fuzzy decision-making information. Presently, BM and HM have been gradually extended to IFSs [31,32], HFSs [33], DHFSs [34,35], PFSs [36], etc. In addition, some scholars have noticed that it is insufficient to only consider the interrelationship among attributes. They realized that DMs usually provided unreasonable evaluation values, which evidently negatively affect the final decision results. Hence, scholars combined the power average (PA) [37] operator with BM and HEM, and proposed the power BM (PBM) [38] and power HEM (PHEM) [39] operators, which are evidently more powerful and useful than PA, BM, and HEM. Due to these reasons, PBM and PHEM have been extensively applied in fusing fuzzy attribute values and quite a few new achievements have been reported [40,41,42,43,44,45]. Recently, by combining PA with Hamy mean (HM) [46], power Hamy mean (PHM) [47], which is more efficient as it has the capability of capturing the interrelationship among multiple attributes. Hence, it is unceasingly worth to studying PHM in solving practical MADM. We provide Table 1 to better demonstrate the development fuzzy sets theories and aggregation operators.

Because of the extreme complexity of real decision-making problems, the above-mentioned decision-making methods based on q-RDHFSs still have limitations. Therefore, the purpose of this paper can be summarized as three points. First, to reduce the bad influence of unreasonable or extreme q-RDHFEs, it is necessary to construct a model to eliminate the influence of extreme values. Therefore, this paper proposed new aggregation operators to fuse q-RDHFEs. Second, when DMs are uncertain about the importance of attributes, to determine the reasonable attribute weights, we consider developing an entropy measure for q-RDHFSs, thereby expanding the application scenarios of this method. Third, to prove the practical value of this method, medical decision-making issues, such as the assessment of hospital medical quality, can be solved by the proposed MADM method.

## 3. Preliminaries

Some basic notions that will be used in the following sections are reviewed in this section.

### 3.1. The q-Rung Dual Hesitant Fuzzy Sets

**Definition** **1** (**[30]**). *Let X be an ordinary fixed set, a q-rung dual hesitant fuzzy set (q-RDHFS) A defined on X is expressed as*

(1) $$A=\left\{\langle x,h_{A}\left(x\right),g_{A}\left(x\right)\rangle \left|x\in X\right.\right\},$$

*where* $h_{A}\left(x\right)$ *and* $g_{A}\left(x\right)$ *are two sets of some interval values in [0, 1], denoting the MD and NMD of the element* x∈X *to the set A, like that* 0≤γ,η≤1 *and* $\gamma^{q}+\eta^{q}\leq 1\left(q\geq 1\right)$*, where* $\gamma \in h_{A}\left(x\right)$ *and* $\eta \in g_{A}\left(x\right)$ *for all* x∈X*. For convenience, the ordered pair* $d_{A}\left(x\right)=\left(h_{A}\left(x\right),g_{A}\left(x\right)\right)$ *is called a q-rung dual hesitant fuzzy element (q-RDHFE), which can be symbolized as* $d=\left(h,g\right)$ *for simplicity.*

*Xu et al.* [17] *proposed the operations of q-RDHFEs.*

**Definition** **2** (**[17]**). *Let* $d_{1}=\left(h_{1},g_{1}\right)$*,* $d_{2}=\left(h_{2},g_{2}\right)$ *and* $d=\left(h,g\right)$ *be any three q-RDHFEs, and* λ *be a positive real number, then*

(1) $d_{1}⊕d_{2}=\cup_{\gamma_{1}\in h_{1},\gamma_{2}\in h_{2},\eta_{1}\in g_{1},\eta_{2}\in g_{2}}\left\{\left\{{\left(\gamma_{1}^{q}+\gamma_{2}^{q}-\gamma_{1}^{q}\gamma_{2}^{q}\right)}^{1/q}\right\},\left\{\eta_{1}\eta_{2}\right\}\right\}$;
(2) $d_{1}⊗d_{2}=\cup_{\gamma_{1}\in h_{1},\gamma_{2}\in h_{2},\eta_{1}\in g_{1},\eta_{2}\in g_{2}}\left\{\left\{\gamma_{1}\gamma_{2}\right\},\left\{{\left(\eta_{1}^{q}+\eta_{2}^{q}-\eta_{1}^{q}\eta_{2}^{q}\right)}^{1/q}\right\}\right\}$;
(3) $\lambda d=\cup_{\gamma \in h,\eta \in g}\left\{\left\{{\left(1-{\left(1-\gamma^{q}\right)}^{\lambda }\right)}^{1/q}\right\},\left\{\eta^{\lambda }\right\}\right\}$;
(4) $d^{\lambda }=\cup_{\gamma \in h,\eta \in g}\left\{\left\{\gamma^{\lambda }\right\},\left\{{\left(1-{\left(1-\eta^{q}\right)}^{\lambda }\right)}^{1/q}\right\}\right\}$.

*Xu et al.* [17] *presented a comparison method to sort any q-RDHFEs.*

**Definition** **3** (**[17]**). *Let* $d=\left(l,p\right)$ *be a q-RDHFE, then the score function of* ε *is defined as*

(2) $$S\left(d\right)={\left(\frac{1}{\#l}\sum_{\gamma \in l}\gamma \right)}^{q}-{\left(\frac{1}{\#p}\sum_{\eta \in p}\eta \right)}^{q},$$

 *and the accuracy function of* ε *is defined as*

(3) $$H\left(d\right)={\left(\frac{1}{\#l}\sum_{\gamma \in l}\gamma \right)}^{q}+{\left(\frac{1}{\#p}\sum_{\eta \in p}\eta \right)}^{q},$$

*Let* $d_{1}=\left(l_{1},p_{1}\right)$ *and* $d_{2}=\left(l_{2},p_{2}\right)$ *be any two q-RDHFEs, then *

(1) *If * $S\left(d_{1}\right)>S\left(d_{2}\right)$*, then* $d_{1}>d_{2}$;
(2) *If * $S\left(d_{1}\right)=S\left(d_{2}\right)$, *then*
  *if * $H\left(d_{1}\right)>H\left(d_{2}\right)$*, then* $d_{1}>d_{2}$*;*
  *if * $H\left(d_{1}\right)=H\left(d_{2}\right)$*, then* $d_{1}=d_{2}$*;*

*Then, we introduce the distance measure between any two q-RDHFEs.*

**Definition** **4.** *Let* $d_{1}=\left(h_{1},g_{1}\right)$ *and* $d_{2}=\left(h_{2},g_{2}\right)$ *be two q-RDHFEs, then the distance measure between* $d_{1}$ *and* $d_{2}$ *is defined as*

(4) $$d\left(d_{1},d_{2}\right)=\left(\frac{1}{\left(\#h+\#g\right)}\left(\sum_{i=1}^{\#h}\left|{\left({\left(\gamma_{1}\right)}_{\sigma \left(i\right)}\right)}^{q}-{\left({\left(\gamma_{2}\right)}_{\sigma \left(i\right)}\right)}^{q}\right|+\sum_{j=1}^{\#g}\left|{\left({\left(\eta_{1}\right)}_{\sigma \left(j\right)}\right)}^{q}-{\left({\left(\eta_{2}\right)}_{\sigma \left(j\right)}\right)}^{q}\right|\right)\right),$$

*where*${\left(\gamma_{1}\right)}_{\sigma \left(i\right)}\in h_{1}$*,*${\left(\gamma_{2}\right)}_{\sigma \left(i\right)}\in h_{2}$*,*${\left(\eta_{1}\right)}_{\sigma \left(j\right)}\in g_{1}$*,*${\left(\eta_{2}\right)}_{\sigma \left(j\right)}\in g_{2}$. #h *as a sign of the number of elements in*$h_{1}$ *and*$h_{2}$*, and*#g *symbolize the number of elements in*$g_{1}$ *and*$g_{2}$.

**Remark** **1.** *Let* $d_{1}=\left(h_{1},g_{1}\right)$ *and* $d_{2}=\left(h_{2},g_{2}\right)$ *be any two q-RDHFEs. From Definition 4, it should be noticed that* $h_{1}$ *and* $h_{2}$ *should have the same number of values, and* $g_{1}$ *and* $g_{2}$ *are supposed to have the same number of values when computing the distance. Nonetheless, this situation will not always be satisfied. Therefore, in order to calculate accurately, the shorter q-RDHFE is supposed to adding values to ensure that the number of MDs and NMDs of the two q-RDHFEs is equal. Then some supplementary rules are proposed for the shorter q-RDHFE.*

Let

$$d_{1}=\left(h_{1},g_{1}\right)=\left\{\begin{array}{l} \left\{{\left(\gamma_{1}\right)}_{\sigma \left(1\right)},{\left(\gamma_{1}\right)}_{\sigma \left(2\right)},\ldots ,{\left(\gamma_{1}\right)}_{\sigma \left(\#h_{1}\right)}\right\}\\ \left\{{\left(\eta_{1}\right)}_{\sigma \left(1\right)},{\left(\eta_{1}\right)}_{\sigma \left(2\right)},\ldots ,{\left(\eta_{1}\right)}_{\sigma \left(\#g_{1}\right)}\right\} \end{array}\right\},$$

and

$$d_{2}=\left(h_{2},g_{2}\right)=\left\{\begin{array}{l} \left\{{\left(\gamma_{2}\right)}_{\sigma \left(1\right)},{\left(\gamma_{2}\right)}_{\sigma \left(2\right)},\ldots ,{\left(\gamma_{2}\right)}_{\sigma \left(\#h_{2}\right)}\right\}\\ \left\{{\left(\eta_{2}\right)}_{\sigma \left(1\right)},{\left(\eta_{2}\right)}_{\sigma \left(2\right)},\ldots ,{\left(\eta_{2}\right)}_{\sigma \left(\#g_{2}\right)}\right\} \end{array}\right\},$$

If $\#h_{1}<\#h_{2}$ and $\#g_{1}>\#g_{2}$, there are two methods to supplement $d_{1}$ and $d_{2}$. When DMs are optimistic, the method to extend $d_{1}$ and $d_{2}$ to $d_{1}{}^{\prime }$ and $d_{2}{}^{\prime }$ is adding the largest values in $h_{1}$ and $g_{2}$. On the contrary, if DMs have pessimistic evaluations, the method is that add the smallest values in $h_{1}$ and $g_{2}$. For convenience, we suppose DMs are optimistic in our paper and the first method is taken to supplement shorter q-RDHFEs.

**Example** **1.** *Let two q-RDHFEs are* $d_{1}=\left\{\left\{0.3\right\},\left\{0.5,0.7,0.8\right\}\right\}$ *and* $d_{2}=\left\{\left\{0.2,0.4\right\},\left\{0.5,0.8\right\}\right\}$*. For calculation,* $d_{1}$ *and* $d_{2}$ *could be changed into* $d_{1}^{\prime }$ *and* $d_{2}^{\prime }$ *, specifically (*q=6*)*.

$$d_{1}^{\prime }=\left\{\left\{0.3,0.3\right\},\left\{0.5,0.7,0.8\right\}\right\},$$

$$d_{2}^{\prime }=\left\{\left\{0.2,0.4\right\},\left\{0.5,0.8,0.8\right\}\right\}.$$

Then,

$$d\left(d_{1},d_{2}\right)=\left(\frac{1}{\left(2+3\right)}\left(\left(\left|{\left(0.3\right)}^{q}-{\left(0.2\right)}^{q}\right|+\left|{\left(0.3\right)}^{q}-{\left(0.4\right)}^{q}\right|\right)+\left(\left|{\left(0.5\right)}^{q}-{\left(0.5\right)}^{q}\right|+\left|{\left(0.7\right)}^{q}-{\left(0.8\right)}^{q}\right|+\left|{\left(0.8\right)}^{q}-{\left(0.8\right)}^{q}\right|\right)\right)\right)$$

$$=\left(\frac{1}{5}\left({\left(0.4\right)}^{q}-{\left(0.2\right)}^{q}+{\left(0.8\right)}^{q}-{\left(0.7\right)}^{q}\right)\right)=0.0297$$

For two q-RDHFEs $d_{1}$ and $d_{2}$, the distance between $d_{1}$ and $d_{2}$, symbolized as $d\left(d_{1},d_{2}\right)$, should satisfy the following properties:

(1) $0\leq d\left(d_{1},d_{2}\right)\leq 1$;
(2) $d\left(d_{1},d_{2}\right)=0$ if and only if $d_{1}=d_{2}$;
(3) $d\left(d_{1},d_{2}\right)=d\left(d_{2},d_{1}\right)$.

### 3.2. PA, HM and PHM Operators

**Definition** **5** (**[37]**). *Let* $a_{i}\left(i=1,2,\ldots ,n\right)$ *be a collection of non-negative crisp numbers, then the PA operator is defined as*

(5) $$PA\left(a_{1},a_{2},\ldots ,a_{n}\right)=\frac{\sum_{i=1}^{n}\left(1+T\left(a_{i}\right)\right)a_{i}}{\sum_{i=1}^{n}\left(1+T\left(a_{i}\right)\right)},$$

*where* $T\left(a_{i}\right)=\sum_{j=1,i\neq j}^{n}Sup\left(a_{i},a_{j}\right)$ *,* $Sup\left(a_{i},a_{j}\right)$ *symbolizes the support for* $a_{i}$ *from* $a_{j}$ *, satisfying the conditions:*

(1) $0\leq Sup\left(a_{i},a_{j}\right)\leq 1$
(2) $Sup\left(a_{i},a_{j}\right)=Sup\left(a_{j},a_{i}\right)$;
(3) $Sup\left(a,b\right)\leq Sup\left(c,d\right)$*, if* $\left|a,b\right|\geq \left|c,d\right|$.

**Definition** **6** **[46]. ***Let* $a_{i}\left(i=1,2,\ldots ,n\right)$ *be a collection of nonnegative real numbers, and* k=1,2,…,n*. If*

(6) $$HM^{\left(k\right)}\left(a_{1},a_{2},\ldots ,a_{n}\right)=\frac{\sum_{1\leq i_{1}<\cdots <i_{k}\leq n}{\left(\prod_{j=1}^{k}a_{i_{j}}\right)}^{1/k}}{C_{n}^{k}},$$

*Then*$HM^{\left(k\right)}$ *is the HM operator, where*$\left(i_{1},i_{2},\ldots ,i_{k}\right)$ *traverses all the k-tuple combination of*$\left(1,2,\ldots ,n\right)$ *and*$C_{n}^{k}$ *is the binomial coefficient.*

**Definition** **7.** *Let* $a_{i}\left(i=1,2,\ldots ,n\right)$ *be a collection of nonnegative real numbers, and* k=1,2,…,n*. The power Hamy mean (PHM) operator is defined as*

(7) $$PHM^{\left(k\right)}\left(a_{1},a_{2},\ldots ,a_{n}\right)=\frac{1}{C_{n}^{k}}\left(\sum_{1\leq i_{1}<\cdots <i_{k}\leq n}{\left(\prod_{j=1}^{k}\left(n\frac{\left(1+T\left(a_{i_{j}}\right)\right)a_{i_{j}}}{\sum_{j=1}^{n}\left(1+T\left(a_{j}\right)\right)}\right)\right)}^{1/k}\right),$$

*where* $\left(i_{1},i_{2},\ldots ,i_{k}\right)$ *traverses all the k-tuple combination of* $\left(1,2,\ldots ,n\right)$ *and* $C_{n}^{k}$ *is the binomial coefficient.* $T\left(a_{i}\right)=\sum_{j=1,i\neq j}^{n}Sup\left(a_{i},a_{j}\right)$*,* $Sup\left(a_{i},a_{j}\right)$ *symbolize support value for* $a_{i}$ *from* $a_{j}$*, satisfying the properties presented in Definition 5.*

## 4. Some Aggregation Operators and Their Properties

We extend the powerful PHM to q-RDHFEs and discuss their properties in this section.

### 4.1. The q-Rung Dual Hesitant Fuzzy Power Hamy Mean Operator

**Definition** **8.** *Let* $d_{i}\left(i=1,2,\ldots ,n\right)$ *is a collection of q-RDHFEs and* k=1,2,…,n*. The q-rung dual hesitant fuzzy power Hamy mean (q-RDHFPHM) operator is as follows*

(8) $$q-RDHFPHM^{\left(k\right)}\left(d_{1},d_{2},\ldots ,d_{n}\right)=\frac{1}{C_{n}^{k}}\left(\underset{1\leq i_{1}<\cdots <i_{k}\leq n}{⊕}{\left(\overset{k}{\underset{j=1}{⊗}}\left(n\frac{\left(1+T\left(d_{i_{j}}\right)\right)d_{i_{j}}}{\sum_{i=1}^{n}\left(1+T\left(d_{i}\right)\right)}\right)\right)}^{1/k}\right),$$

*where* $\left(i_{1},i_{2},\ldots ,i_{k}\right)$ * traverses all the k-tuple combination of* $\left(1,2,\ldots ,n\right)$ *and* $C_{n}^{k}$ * is the binomial coefficient.* $T\left(d_{i_{j}}\right)=\sum_{j=1,i\neq j}^{n}Sup\left(d_{i},d_{j}\right)$*, which should be satisfied following properties:*

(1) $0\leq Sup\left(d_{i},d_{j}\right)\leq 1$;
(2) $Sup\left(d_{i},d_{j}\right)=Sup\left(d_{j},d_{i}\right)$;
(3) $Sup\left(d_{i},d_{j}\right)\leq Sup\left(d_{s},d_{t}\right)$, if $dis\left(d_{i},d_{j}\right)\geq dis\left(d_{s},d_{t}\right)$, and $dis\left(d_{i},d_{j}\right)$ is the distance between $d_{i}$ and $d_{j}$.

*If we assume*

(9) $$\delta_{i}=\frac{1+T\left(d_{i}\right)}{\sum_{i=1}^{n}\left(1+T\left(d_{i}\right)\right)},$$

 *then Equation (8) can be transformed into the following form*

(10) $$q-RDHFPHM^{\left(k\right)}\left(d_{1},d_{2},\ldots ,d_{n}\right)=\frac{1}{C_{n}^{k}}\left(\underset{1\leq i_{1}<\cdots <i_{k}\leq n}{⊕}{\left(\overset{k}{\underset{j=1}{⊗}}\left(n\delta_{i_{j}}d_{i_{j}}\right)\right)}^{1/k}\right),$$

*where*$\delta ={\left(\delta_{1},\delta_{2},\ldots ,\delta_{n}\right)}^{T}$ *is called the power weight vector, such that*$0\leq \delta_{i}\leq 1$ *and*$\sum_{i=1}^{n}\delta_{i}=1$.

**Theorem** **1.** *Let* $d_{i}=\left(h_{i},g_{i}\right)\left(i=1,2,\ldots ,n\right)$ *be a series of q-RDHFEs, the aggregated value by the q-RDHFPHM operator is still a q-RDHFE and*

$$q-RDHFPHM^{\left(k\right)}\left(d_{1},d_{2},\ldots ,d_{n}\right)=\cup_{\gamma_{i_{j}}\in h_{i_{j}},\eta_{i_{j}}\in g_{i_{j}}}$$

(11) $$\left\{\left\{{\left(1-{\prod_{1\leq i_{1}<\ldots <i_{k}\leq n}\left(1-\prod_{j=1}^{k}{\left(1-{\left(1-\gamma_{i_{j}}^{q}\right)}^{n\delta_{i_{j}}}\right)}^{\frac{1}{k}}\right)}^{{}^{\frac{1}{C_{n}^{k}}}}\right)}^{\frac{1}{q}}\right\},\left\{\prod_{1\leq i_{1}<\ldots <i_{k}\leq n}{\left(1-{\prod_{j=1}^{k}\left(1-\eta_{i_{j}}^{nq\delta_{i_{j}}}\right)}^{\frac{1}{k}}\right)}^{\frac{1}{qC_{n}^{k}}}\right\}\right\}.$$

**Proof.**  See Appendix A. □

**Theorem** **2.** *(Idempotency): Let* $d_{i}=\left(h_{i},g_{i}\right)\left(i=1,2,\ldots ,n\right)$ *be a collection of q-RDHFEs, if* $d_{i}=d=\left(h,g\right)$ *for all i, then*

(12) $$q-RDHFPHM^{\left(k\right)}\left(d_{1},d_{2},\ldots ,d_{n}\right)=d.$$

**Proof.**  See Appendix B. □

**Theorem** **3.** *(Boundedness): Let* $d_{i}=\left(h_{i},g_{i}\right)\left(i=1,2,\ldots ,n\right)$ *is a set of q-RDHFEs,* $d^{-}=\min\left(d_{1},d_{2},\ldots ,d_{n}\right)$ *and* $d^{+}=\max\left(d_{1},d_{2},\ldots ,d_{n}\right)$*, then *

(13) $$x\leq q-RDHFPHM^{\left(k\right)}\left(d_{1},d_{2},\ldots ,d_{n}\right)\leq y.$$

where $x=\frac{1}{C_{n}^{k}}\left(\underset{1\leq i_{1}<\cdots <i_{k}\leq n}{⊕}{\left(\overset{k}{\underset{j=1}{⊗}}\left(n\delta_{i_{j}}d^{-}\right)\right)}^{1/k}\right)$ and $y=\frac{1}{C_{n}^{k}}\left(\underset{1\leq i_{1}<\cdots <i_{k}\leq n}{⊕}{\left(\overset{k}{\underset{j=1}{⊗}}\left(n\delta_{i_{j}}d^{+}\right)\right)}^{1/k}\right)$.

**Proof.**  See Appendix C. □

Then, some special cases of the proposed q-RDHFPHM operator with respect to *q* and *k* will be inferenced.

**Case** **1.** *If*k=1 *, the q-RDHFPHM operator is reduced to the q-rung dual hesitant fuzzy power average (q-RDHFPA) operator*.

(14) $$q-RDHFPHM^{\left(k\right)}\left(d_{1},d_{2},\ldots ,d_{n}\right)=\overset{n}{\underset{i=1}{⊕}}\delta_{i}d_{i}=\cup_{\gamma_{i}\in h_{i},\eta_{i}\in g_{i}}\left\{\left\{{\left(1-\prod_{i=1}^{n}{\left(1-\gamma_{i}^{q}\right)}^{\delta_{i}}\right)}^{\frac{1}{q}}\right\},\left\{\prod_{i=1}^{n}\eta_{i}^{\delta_{i}}\right\}\right\}.$$

Besides, when $Sup\left(d_{i},d_{j}\right)=t>0$, a q-RDHFPHM operator is reduced to a q-rung dual hesitant fuzzy average (q-RDHFA) operator.

(15) $$q-RDHFPHM^{\left(k\right)}\left(d_{1},d_{2},\ldots ,d_{n}\right)=\frac{1}{n}\overset{n}{\underset{i=1}{⊕}}d_{i}=\cup_{\gamma_{i}\in h_{i},\eta_{i}\in g_{i}}\left\{\left\{{\left(1-\prod_{i=1}^{n}{\left(1-\gamma_{i}^{q}\right)}^{\frac{1}{n}}\right)}^{\frac{1}{q}}\right\},\left\{\prod_{i=1}^{n}\eta_{i}^{\frac{1}{n}}\right\}\right\}.$$

**Case** **2.** *If* q=1*, the q-RDHFPHM operator is reduced to the dual hesitant fuzzy power Hamy mean (DHFPHM) operator*.

$$q-RDHFPHM^{\left(k\right)}\left(d_{1},d_{2},\ldots ,d_{n}\right)=\cup_{\gamma_{i_{j}}\in h_{i_{j}},\eta_{i_{j}}\in g_{i_{j}}}$$

(16) $$\left\{\left\{1-{\prod_{1\leq i_{1}<\ldots <i_{k}\leq n}\left(1-\prod_{j=1}^{k}{\left(1-{\left(1-\gamma_{i_{j}}\right)}^{n\delta_{i_{j}}}\right)}^{\frac{1}{k}}\right)}^{{}^{\frac{1}{C_{n}^{k}}}}\right\},\left\{\prod_{1\leq i_{1}<\ldots <i_{k}\leq n}{\left(1-{\prod_{j=1}^{k}\left(1-\eta_{i_{j}}^{n\delta_{i_{j}}}\right)}^{\frac{1}{k}}\right)}^{\frac{1}{C_{n}^{k}}}\right\}\right\}.$$

Besides, if $Sup\left(d_{i},d_{j}\right)=t>0$, the q-RDHFPHM operator is reduced to the dual hesitant fuzzy Hamy mean (DHFHM) operator.

$$q-RDHFPHM^{\left(k\right)}\left(d_{1},d_{2},\ldots ,d_{n}\right)=\cup_{\gamma_{i_{j}}\in h_{i_{j}},\eta_{i_{j}}\in g_{i_{j}}}$$

(17) $$\left\{\left\{1-{\prod_{1\leq i_{1}<\ldots <i_{k}\leq n}\left(1-\prod_{j=1}^{k}{\left(\gamma_{i_{j}}\right)}^{\frac{1}{k}}\right)}^{{}^{\frac{1}{C_{n}^{k}}}}\right\},\left\{\prod_{1\leq i_{1}<\ldots <i_{k}\leq n}{\left(1-{\prod_{j=1}^{k}\left(1-\eta_{i_{j}}\right)}^{\frac{1}{k}}\right)}^{\frac{1}{C_{n}^{k}}}\right\}\right\}.$$

**Case** **3.** *When*q=2*, the q-RDHFPHM operator is reduced to the dual hesitant Pythagorean fuzzy power Hamy mean (DHPFPHM) operator*.

$$q-RDHFPHM^{\left(k\right)}\left(d_{1},d_{2},\ldots ,d_{n}\right)=\cup_{\gamma_{i_{j}}\in h_{i_{j}},\eta_{i_{j}}\in g_{i_{j}}}$$

(18) $$\left\{\left\{{\left(1-{\prod_{1\leq i_{1}<\ldots <i_{k}\leq n}\left(1-\prod_{j=1}^{k}{\left(1-{\left(1-\gamma_{i_{j}}^{2}\right)}^{n\delta_{i_{j}}}\right)}^{\frac{1}{k}}\right)}^{{}^{\frac{1}{C_{n}^{k}}}}\right)}^{\frac{1}{2}}\right\},\left\{\prod_{1\leq i_{1}<\ldots <i_{k}\leq n}{\left(1-{\prod_{j=1}^{k}\left(1-\eta_{i_{j}}^{2n\delta_{i_{j}}}\right)}^{\frac{1}{k}}\right)}^{\frac{1}{2C_{n}^{k}}}\right\}\right\}.$$

Besides, if $Sup\left(d_{i},d_{j}\right)=t>0$, the q-RDHFPHM operator is reduced to the DHPFPHM operator.

$$q-RDHFPHM^{\left(k\right)}\left(d_{1},d_{2},\ldots ,d_{n}\right)=\cup_{\gamma_{i_{j}}\in h_{i_{j}},\eta_{i_{j}}\in g_{i_{j}}}$$

(19) $$\left\{\left\{{\left(1-{\prod_{1\leq i_{1}<\ldots <i_{k}\leq n}\left(1-\prod_{j=1}^{k}{\left(\gamma_{i_{j}}^{2}\right)}^{\frac{1}{k}}\right)}^{{}^{\frac{1}{C_{n}^{k}}}}\right)}^{\frac{1}{2}}\right\},\left\{\prod_{1\leq i_{1}<\ldots <i_{k}\leq n}{\left(1-{\prod_{j=1}^{k}\left(1-\eta_{i_{j}}^{2}\right)}^{\frac{1}{k}}\right)}^{\frac{1}{2C_{n}^{k}}}\right\}\right\}.$$

**Case** **4.** *When*k=n*, a q-RDHFPHM operator is reduced to a q-rung dual hesitant fuzzy power geometric (q-RDHFPG) operator*.

$$q-RDHFPHM^{\left(k\right)}\left(d_{1},d_{2},\ldots ,d_{n}\right)={\left(\overset{n}{\underset{j=1}{⊗}}n\delta_{j}d_{j}\right)}^{\frac{1}{n}}$$

(20) $$=\cup_{\gamma_{j}\in h_{j},\eta_{j}\in g_{j}}\left\{\left\{{\prod_{j=1}^{n}\left(1-{\left(1-\gamma_{j}^{q}\right)}^{n\delta_{j}}\right)}^{\frac{1}{nq}}\right\},\left\{{\left(1-\prod_{j=1}^{n}{\left(1-\eta_{j}^{nq\delta_{j}}\right)}^{\frac{1}{n}}\right)}^{\frac{1}{q}}\right\}\right\}.$$

Besides, when $Sup\left(d_{i},d_{j}\right)=t>0$, a q-RDHFPHM operator is reduced to a q-rung dual hesitant fuzzy geometric (q-RDHFG) operator.

(21) $$q-RDHFPHM^{\left(k\right)}\left(d_{1},d_{2},\ldots ,d_{n}\right)=\overset{n}{\underset{j=1}{⊗}}d_{j}^{1/n}=\cup_{\gamma_{j}\in h_{j},\eta_{j}\in g_{j}}\left\{\left\{\prod_{j=1}^{n}\gamma_{j}^{\frac{1}{n}}\right\},\left\{{\left(1-{\prod_{j=1}^{n}\left(1-\eta_{j}^{q}\right)}^{\frac{1}{n}}\right)}^{\frac{1}{q}}\right\}\right\}.$$

**Remark** **2.** *More special cases of the q-RDHFPHM operator can be obtained. For example, if*q=k=1*, then the q-RDHFPHM operator reduces to the dual hesitant fuzzy power average operator. If* q=1 *and* k=2*, then the q-RDHFPHM operator reduces to the dual hesitant fuzzy power geometric mean operator. Some other aggregation operators, such as dual hesitant Pythagorean fuzzy power average operator, and dual hesitant Pythagorean fuzzy power geometric operator*.

### 4.2. The q-Rung Dual Hesitant Fuzzy Power Weighted Hamy Mean Operator

**Definition** **9.** *Let* $d_{i}\left(i=1,2,\ldots ,n\right)$ *is a collection of q-RDHFEs and* k=1,2,…,n*. Let* $w={\left(w_{1},w_{2},\ldots ,w_{n}\right)}^{T}$ *is a weight vector, satisfying* $0\leq w_{i}\leq 1$ *and* $\sum_{i=1}^{n}w_{i}=1$*. The q-rung dual hesitant fuzzy power weighted Hamy mean (q-RDHFPWHM) operator is defined as*

(22) $$q-RDHFPWHM^{\left(k\right)}\left(d_{1},d_{2},\ldots ,d_{n}\right)=\frac{1}{C_{n}^{k}}\left(\underset{1\leq i_{1}<\cdots <i_{k}\leq n}{⊕}{\left(\overset{k}{\underset{j=1}{⊗}}\left(n\frac{w_{i_{j}}\left(1+T\left(d_{i_{j}}\right)\right)d_{i_{j}}}{\sum_{i=1}^{n}w_{i}\left(1+T\left(d_{i}\right)\right)}\right)\right)}^{1/k}\right),$$

*where* $\left(i_{1},i_{2},\ldots ,i_{k}\right)$ *traverses all the k-tuple combination of* $\left(1,2,\ldots ,n\right)$ *and* $C_{n}^{k}$ *is the binomial coefficient.* $T\left(d_{i}\right)=\sum_{j=1,i\neq j}^{n}Sup\left(d_{i},d_{j}\right)$*, which also satisfying the properties presented in Definition 7. If we assume*

(23) $$\sigma_{i}=\frac{w_{i}\left(1+T\left(d_{i}\right)\right)}{\sum_{i=1}^{n}w_{i}\left(1+T\left(d_{i}\right)\right)},$$

 *then we can rewrite Equation (22) as*

(24) $$q-RDHFPWHM^{\left(k\right)}\left(d_{1},d_{2},\ldots ,d_{n}\right)=\frac{1}{C_{n}^{k}}\left(\underset{1\leq i_{1}<\cdots <i_{k}\leq n}{⊕}{\left(\overset{k}{\underset{j=1}{⊗}}\left(n\sigma_{i_{j}}d_{i_{j}}\right)\right)}^{1/k}\right),$$

*where*$\sigma ={\left(\sigma_{1},\sigma_{2},\ldots ,\sigma_{n}\right)}^{T}$ *is known as the power weight vector*$0\leq \sigma_{i}\leq 1$ *and*$\sum_{i=1}^{n}\sigma_{i}=1$.

*Based on the operational rules of q-RDHFEs, the aggregated result of q-RDHFPWHM operator is derived.*

**Theorem** **4.** *Let* $d_{i}=\left(h_{i},g_{i}\right)\left(i=1,2,\ldots ,n\right)$ *be a series of q-RDHFEs, the aggregated value by the q-RDHFPWHM operator is still a q-RDHFE and*

$$q-RDHFPWHM^{\left(k\right)}\left(d_{1},d_{2},\ldots ,d_{n}\right)=$$

(25) $$\cup_{\gamma_{i_{j}}\in h_{i_{j}},\eta_{i_{j}}\in g_{i_{j}}}\left\{\left\{{\left(1-{\prod_{1\leq i_{1}<\ldots <i_{k}\leq n}\left(1-\prod_{j=1}^{k}{\left(1-{\left(1-\gamma_{i_{j}}^{q}\right)}^{n\sigma_{i_{j}}}\right)}^{\frac{1}{k}}\right)}^{{}^{\frac{1}{C_{n}^{k}}}}\right)}^{\frac{1}{q}}\right\},\left\{\prod_{1\leq i_{1}<\ldots <i_{k}\leq n}{\left(1-{\prod_{j=1}^{k}\left(1-\eta_{i_{j}}^{nq\sigma_{i_{j}}}\right)}^{\frac{1}{k}}\right)}^{\frac{1}{qC_{n}^{k}}}\right\}\right\}.$$

**Proof.**  See Appendix D. □

**Theorem** **5.** *(Boundedness): Let* $d_{i}=\left(h_{i},g_{i}\right)\left(i=1,2,\ldots ,n\right)$ *be a collection of q-RDHFEs,* $d^{-}=\min\left(d_{1},d_{2},\ldots ,d_{n}\right)$ *and* $d^{+}=\max\left(d_{1},d_{2},\ldots ,d_{n}\right)$*, then*

(26) $$x\leq q-RDHFPWHM^{\left(k\right)}\left(d_{1},d_{2},\ldots ,d_{n}\right)\leq y.$$

*where* $x=\frac{1}{C_{n}^{k}}\left(\underset{1\leq i_{1}<\cdots <i_{k}\leq n}{⊕}{\left(\overset{k}{\underset{j=1}{⊗}}\left(n\sigma_{i_{j}}d^{-}\right)\right)}^{1/k}\right)$ and $y=\frac{1}{C_{n}^{k}}\left(\underset{1\leq i_{1}<\cdots <i_{k}\leq n}{⊕}{\left(\overset{k}{\underset{j=1}{⊗}}\left(n\sigma_{i_{j}}d^{+}\right)\right)}^{1/k}\right)$.

**Proof.**  See Appendix E. □

## 5. A Method to Determine the Attribute Weights Based on Entropy

Entropy is a widely used tool to measure the uncertainties in fuzzy sets theory. In addition, in quite a few practical MADM problems, the weight vector of attributes is completely unknown. It is widely acknowledged by DMs that such attribute vector plays an important role in MADM problems [48,49,50,51,52]. Hence, before determining the optimal alternatives, the weight information of attributes should be calculated by some methods. Entropy measure is widely accepted as an approach to determine the weights of attributes. Hence, in the followings, we develop an entropy measure for q-RDHFSs and based on which, a method to determine weight information of attributes is proposed. The axiom for entropy measure of q-RDHFEs is presented as follows.

**Definition** **10.** *Let*$d_{1}=\left(h_{1},g_{1}\right)$ *and*$d_{2}=\left(h_{2},g_{2}\right)$ *be any two q-RDHFEs. A function E is an entropy on q-RDHFEs, if and only if E satisfies the following properties.*

(1) $E\left(d_{1}\right)=0$*, if and only if* $d_{1}=\left\{\left\{0\right\},\left\{1\right\}\right\}$ *or*$d_{2}=\left\{\left\{1\right\},\left\{0\right\}\right\}$;
(2) $E\left(d_{1}\right)=1$*, if and only if*$\#h_{1}=\#g_{1}$ *and*${\left(\gamma_{1}\right)}_{\sigma \left(i\right)}={\left(\eta_{1}\right)}_{\sigma \left(i\right)}\left(i=1,2,\ldots ,m\right)$, *where*${\left(\gamma_{1}\right)}_{\sigma \left(i\right)}$ *and*${\left(\eta_{1}\right)}_{\sigma \left(i\right)}$ *are the ith smallest values of*$h_{1}$ *and*$g_{1}$*, respectively;*
(3) $E\left(\rho \right)\leq E\left(\theta \right)$ *if*$max_{i}h_{i}^{1}\leq min_{s}h_{s}^{2}$, $min_{j}g_{i}$
(4)
$E\left(d_{1}\right)=E\left(d_{1}^{C}\right)$

*Based on the axiom, in what follows, we present an entropy measure of q-RDHFE. Let* $d=\left(h,g\right)$ *be a q-RDHFE, then the entropy measure of d is defined as*

(27) $$E\left(d\right)=1-dis\left(d,d^{C}\right)$$

*where*$dis\left(d,d^{C}\right)$ *is distance measure between*d *and its complement*$\rho^{C}$.

**Remark** **3.** *If* $d=\left\{\left\{0.2,0.5,0.6\right\},\left\{0.3,0.7\right\}\right\}$*, then* $d^{C}=\left\{\left\{0.3,0.7\right\},\left\{0.2,0.5,0.6\right\}\right\}$*. In addition,* d *and* $d^{C}$ *should be changed into* $d_{1}^{\prime }$ *and* $d_{2}^{\prime }$*, Particularly (*q=6*)*.

$$d_{1}^{\prime }=\left\{\left\{0.2,0.5,0.6\right\},\left\{0.3,0.7,0.7\right\}\right\},$$

$$d_{2}^{\prime }=\left\{\left\{0.3,0.7,0.7\right\},\left\{0.2,0.5,0.6\right\}\right\}.$$

Then,

$$E\left(d\right)=1-dis\left(d,d^{C}\right)=1-\left(\frac{1}{\left(3+3\right)}\left(\left(\left|{\left(0.2\right)}^{q}-{\left(0.3\right)}^{q}\right|+\left|{\left(0.5\right)}^{q}-{\left(0.7\right)}^{q}\right|+\left|{\left(0.6\right)}^{q}-{\left(0.7\right)}^{q}\right|\right)+\right.\right.$$

$$\left.\left.\left(\left|{\left(0.3\right)}^{q}-{\left(0.2\right)}^{q}\right|+\left|{\left(0.7\right)}^{q}-{\left(0.5\right)}^{q}\right|+\left|{\left(0.7\right)}^{q}-{\left(0.6\right)}^{q}\right|\right)\right)\right).$$

$$=1-\left(\frac{1}{6}\times \left(4\times {\left(0.7\right)}^{q}+2\times \left({\left(0.3\right)}^{q}-{\left(0.2\right)}^{q}-{\left(0.5\right)}^{q}-{\left(0.6\right)}^{q}\right)\right)\right)=1-0.0579=0.9421.$$

Based on the entropy measure of q-RDHFEs, we present a novel method to determine the weights of aggregated q-RDHFEs. Let $d_{i}\left(i=1,2,\ldots ,n\right)$ be a collection of q-RDHFEs, then weight of $d_{i}$ is given as

(28) $$w_{i}=\frac{1-E\left(d_{i}\right)}{n-\sum_{i=1}^{n}E\left(d_{i}\right)}$$

## 6. A Novel MADM Method Based on q-RDHFEs

In this section, a novel approach to MADM based on q-RDHFEs is proposed. The following is a typical MADM problem which has q-RDHFE assessment information. Suppose that $\left\{A_{1},A_{2},\ldots ,A_{m}\right\}$ is *m* alternatives and the performance of the alternatives under a set of *n* attributes $\left\{C_{1},C_{2},\ldots ,C_{n}\right\}$ is evaluated by the DMs. DMs are required to communicate assessment information by a q-RDHFE $d_{ij}=\left(h_{ij},g_{ij}\right)$ with regard to alternative $A_{i}\left(i=1,2,\ldots ,m\right)$ under attribute $C_{j}\left(j=1,2,\ldots ,n\right)$. Therefore, a decision matrix of q-rung dual hesitant fuzzy can be simplified to $R={\left(d_{ij}\right)}_{m\times n}$. The process of choosing the optimal alternative is presented as follow.

Step 1. Transform the decision matrix. Normally, kinds of attributes should be benefit type or cost type. Therefore, the decision matrix can be standardized by the following method.

(29) $$d_{ij}=\left\{\begin{array}{l} \left(h_{ij},g_{ij}\right)C_{j}\;is\;benefit\;type\\ \left(g_{ij},h_{ij}\right)\;C_{j}\;is\;\cos t\;type \end{array}\right.,$$

Step 2. Calculate the $Sup\left(d_{il},d_{im}\right)$ by

(30) $$Sup\left(d_{il},d_{im}\right)=1-d\left(d_{il},d_{im}\right),$$

satisfying that l,m=1,2,…,n; l≠m

Step 3. Calculate $T\left(d_{ij}\right)$ by

(31) $$T\left(d_{ij}\right)=\sum_{l,m=1,l\neq m}^{n}Sup\left(d_{il},d_{im}\right),$$

Step 4. Calculate the weight of $C_{j}\left(j=1,2,\ldots ,n\right)$ based on the entropy measure of q-RDHFEs as the following formula

(32) $$w_{j}=\frac{1-E\left(d_{j}\right)}{n-\sum_{j=1}^{n}E\left(d_{j}\right)},$$

Step 5. Calculate the power weights $\delta_{ij}$ using below method

(33) $$\delta_{ij}=\frac{w_{i}\left(1+T\left(d_{ij}\right)\right)}{\sum_{i=1}^{n}w_{i}\left(1+T\left(d_{ij}\right)\right)},$$

Step 6. Calculate the evaluation values $d_{i}$ of alternative $A_{i}$ based on the q-RDHFPWHM operator.

(34) $$d_{i}=q-RDHFPWHM^{\left(k\right)}\left(d_{i1},d_{i2},\ldots ,d_{in}\right),$$

Step 7. Using definition 8 to sequence the evaluation values $d_{i}\left(i=1,2,\ldots ,n\right)$.

Step 8. Using the sequence of the overall values to sort alternatives, then choose the best one.

## 7. Assessment Indicator System of Hospital Medical Quality

In the context of hospital’s medical quality evaluation, we propose an evaluation system based on the newly developed AOs. The establishment of the evaluation system is divided in two steps: (1) analyze the evaluation factors; (2) prove the rationality of evaluation factors on the basis of MADM method under q-RDHFEs.

### 7.1. Analysis Evaluation Factors from the Perspective of Patients

Hospital medical quality evaluation involves multiple factors and multiple indicators, including indicators such as medical workload and work efficiency. Through literature search and expert consultation, Lang and Song [53] proposed a comprehensive tertiary hospital’s medical quality evaluation index system, which includes three indicators of work efficiency, medical quality and workload.

#### 7.1.1. Work Efficiency

Work efficiency is the most intuitive factor that affects the quality of medical care in a hospital. It includes the utilization rate of hospital beds, the average hospital stay of patients, the cure rate, and the number of outpatient and emergency patients received by each employee per day.

(1) Utilization rate of hospital beds. It can reflect the ratio between the total number of beds used per day and the total number of existing beds, and reflect the load of hospital beds. In addition, high utilization rate indicates that the use of hospital beds is scientific and reasonable.
(2) Average length of hospital stay. The average hospital stay represents the average length of stay of each discharged patient within a period, which is a comprehensive index for estimating hospital efficiency, medical quality, and technical level.
(3) The number of outpatient and emergency patients received by each employee per day. It can reflect the work efficiency of the hospital staff.

#### 7.1.2. Medical Quality

Medical quality is a key factor affecting the survival and development of a hospital, including the cure rate, the success rate of critically ill rescue, and the satisfaction of nursing services.

(1) Cure rate, improvement rate and mortality rate. These indicators are the link quality indicators in the clinical quality evaluation. The patient’s cure status truly reflects the hospital’s medical quality.
(2) Success rate of critically ill rescue. The rescue success rate of critically ill patients not only reflects the medical quality of the hospital and the technical level of medical staff, but also represents the management level of a hospital.
(3) Satisfaction of nursing service. The patient’s satisfaction with the nursing service of medical staff will affect the doctor-patient relationship and the patient’s satisfaction with the hospital.

#### 7.1.3. Workload

The workload of a hospital can describe the medical quality of the hospital from the side. The workload is mainly composed of two aspects: the number of visits and the number of hospitalizations.

(1) Number of visits. The number of visits is the general term for the total number of visits to the hospital for treatment, including emergency and outpatient.
(2) Number of hospitalizations. In general, there is a certain relationship between the number of visits to the hospital and the number of hospitalizations. As the number of visits increases, the number of hospitalizations also increases. Both of these indicators have an impact on the evaluation of hospital workload.

### 7.2. Establish Medical Quality Evaluation System and Decision Matrix

Based on the analysis of existing evaluation indicators, we have constructed a hospital medical quality evaluation system, as shown in Table 2.

Afterwards, to prove the rationality of evaluation factors on the basis of MADM method under q-RDHFEs, we provide a numerical example.

**Example** **2.** *To select the best medical quality form four hospitals*$A_{i}\left(i=1,2,3,4\right)$*, DMs assess the four hospitals under three attributes* $C_{j}\left(j=1,2,3\right)$*, where C_1_ represents the work efficiency; C_2_ represents the medical quality; and C_3_ represents the workload. DMs are required to evaluate the four alternatives with respect to the three attributes* $C_{j}\left(j=1,2,3\right)$ *by q-RDHFEs and the decision matrix* $A_{i}\left(i=1,2,3,4\right)$ *x* $d_{ij}=\left\{\left\{h_{ij}\right\},\left\{g_{ij}\right\}\right\}$ *is obtained, which is shown in*Table 3.

### 7.3. The Decision-Making Process

The method described in Section 5 is used to determine the best alternative. The calculation process is as follows.

Step 1. Since the attributes are benefit types, the step of standardizing the initial decision matrix can be skipped.

Step 2. Compute the support between $d_{il}$ and $d_{im}$, that is, $Sup\left(d_{il},d_{im}\right)$. The symbol $S^{lm}$ is used to represent the value $Sup\left(d_{il},d_{im}\right)\left(l,m=1,2,3;i=1,2,3,4;l\neq m\right)$. Therefore, the result of calculation is as follow.

$$S^{12}=S^{21}=\left(0.6037,0.7323,0.8654,0.8200\right);$$

$$S^{13}=S^{31}=\left(0.8537,0.6838,0.9813,0.9015\right);$$

$$S^{23}=S^{32}=\left(0.7500,0.9560,0.8766,0.8853\right).$$

Step 3. Compute the support $T\left(d_{ij}\right)$. The symbol $T_{ij}$ is used to symbolize the value $T\left(d_{ij}\right)$, and the result is below

$$T=\left[\begin{array}{c} \begin{array}{ccc} 1.4573 & 1.3537 & 1.6037\\ 1.4161 & 1.6883 & 1.6398\\ 1.8467 & 1.7420 & 1.8579\\ 1.7215 & 1.7053 & 1.7868 \end{array} \end{array}\right]$$

Step 4. Calculate the weight of $C_{j}\left(j=1,2,\ldots ,n\right)$ according to Equation (31). Therefore, the result of calculation is as follow.

$$w_{ij}=\left[\begin{array}{c} \begin{array}{ccc} 0.2025 & 0.6271 & 0.1704\\ 0.3825 & 0.2532 & 0.3643\\ 0.2905 & 0.4946 & 0.2149\\ 0.5669 & 0.1230 & 0.3101 \end{array} \end{array}\right]$$

Step 5. Compute the power weight $\delta_{ij}$ and we can obtain

$$\delta_{ij}=\left[\begin{array}{c} \begin{array}{ccc} 0.2059 & 0.6106 & 0.1836\\ 0.3601 & 0.2652 & 0.3747\\ 0.2957 & 0.4848 & 0.2195\\ 0.5631 & 0.1214 & 0.3155 \end{array} \end{array}\right]$$

Step 6. For alternative $A_{i}\left(i=1,2,3,4\right)$, utilized the q-RDHFPWHM operator to compute the evaluation $d_{i}\left(i=1,2,3,4\right)$ (assume that *k* = 1 and *q* = 3).

Step 7. Compute the score values $S\left(d_{i}\right)\left(i=1,2,3,4\right)$ of the overall evaluation values, and we can get

$$S\left(d_{1}\right)=-0.1117,\;S\left(d_{2}\right)=-0.3535,\;S\left(d_{3}\right)=-0.3806,\;S\left(d_{4}\right)=-0.0675$$

Step 8. According to the score values $S\left(d_{i}\right)\left(i=1,2,3,4\right)$, the ranking orders of the alternatives can be determined, that is $A_{4}>A_{1}>A_{2}>A_{3}$, which directs that $A_{4}$ is the optimal alternative.

### 7.4. Analysis of the Impact of Parameters

One of the most important research aspects of AOs is to check out the influence of parameters. Hence, we also conduct sensitivity analysis of the parameters of the proposed decision-making method.

#### 7.4.1. The Influence of the Parameter *q* on the Results

First of all, the impact of the parameter *q* will be investigated. Hence, different *q* in the process of the calculation (we assume that *k* = 2) are taken and show the results in Table 4. From Table 4, when different parameters of *q* are employed, different score values of alternatives are derived, which may also lead to different ranking results of alternatives. In addition, we also noticed that although the ranking orders are different, the best option is always $A_{4}$. This finding also illustrates the stability of our decision-making method. Moreover, we shall notice that the method of determining the value of *q* is also am important problem. In [30], authors have discussed the method of choosing a proper value of *q*. More details of determining the value of q can be found in Xu et al.’s publication.

#### 7.4.2. The Influence of the Parameter *k* on the Results

The impact of the parameter *k* should be studied in the following. The parameter *k* is a significant parameter in the q-RDHFPWHM operator. If we use different values of the parameter *k*, we can obtain the following decision results, including the score values of alternatives as well as their ranking orders (See Table 5). We noticed that if the values of *k* are different, the score values of alternatives are different, which further lead to slightly different ranking orders of alternatives. However, the optimal alternative is always $A_{4}$. However, it is obvious that the score values according the increase of *k* are smaller. In addition, *k* represents the numbers of attributes among which their interrelationship is taken into consideration. In real MADM problem, DMs can select proper parameter *k* according to practical needs.

### 7.5. Validity Analysis

In this section, we use our method and some existing method include the method proposed by Xu et al. [17] using q-rung dual hesitant fuzzy weighted Heronian mean (q-RDHFWHM) operator, the method proposed by Wei et al. [54] based on dual hesitant Pythagorean fuzzy Hamacher weighted averaging (DHPFHWA) operator and the method proposed by Zhang et al. [55] based on dual hesitant fuzzy Maclaurin symmetric mean (DHFMSM) operator to solve some practical examples and compare their decision results.

#### 7.5.1. Compared with Xu et al.’s Method

In this section, we compare our method with the method proposed by Xu et al. [17] based on q-RDHFWHM operator. The two methods are used to solve the Example 2 and the results are shown in Table 6. As we can see from the Table 6, it is obvious that the score values are different, which leads to different orders. However, the optimal alternative is always $A_{4}$. Hence, it indicates the feasibility of our method.

#### 7.5.2. Compared with Wei et al.’s Method

In this section, we compare our method with the method proposed by Wei et al. [54] based on DHPFHWA operator. We use the two methods to solve the Example 2 and the results are shown in Table 7. From the Table 7, although the score values and ranking orders obtained by different methods are different, the optimal alternative is always $A_{4}$, which illustrate the validity of our method.

#### 7.5.3. Compared with Zhang et al.’s Method

In this subsection, we compare the method presented by Zhang et al. [55] based on dual hesitant fuzzy Maclaurin symmetric mean (DHFMSM) with our method based on q-RDHFPWHM.

**Example** **3.** *An investment company desires to select a city to expand its business. After investigation, there are five cities*$A_{i}\left(i=1,2,3,4,5\right)$ *may be selected. DMs assess the alternatives under four attributes*$C_{j}\left(j=1,2,3,4\right)$*, where*$C_{1}$ *represents resources;*$C_{2}$ *represents politics and policy;*$C_{3}$ *represents economy; and*$C_{4}$ *represents infrastructure. DMs requested to evaluate the five alternatives with respect to the four attributes by q-RDHFEs and the decision matrices is shown in Table 8.*

According to Table 9, different score values of alternatives are computed by two methods. Nevertheless, the ranking orders and the optimal alternative are the same, i.e., $A_{5}>A_{3}>A_{4}>A_{2}>A_{1}$, and $A_{5}$ is the best alternative. In other words, it proves the flexibility of our method.

### 7.6. Advantages of Our Method

In this part, the advantages and superiority of our method are further proved.

#### 7.6.1. It Can Effectively Deal with DMs’ Unreasonable Evaluation Values

Based on the q-RDHFPWHM operator, our method can effectively handle extreme evaluation values, which is demonstrated by the following cases.

**Example** **4.** *In order to state more clearly, we suppose that the DM have personal preferences: the DM are biased against the city*$A_{3}$ *and prefer the city*$A_{5}$ *under city environment*$C_{3}$*. Hence, the DM give*$A_{3}$ *a low evaluation*$\left\{\left\{0.1,0.2,0.3\right\},\left\{0.1,0.2\right\}\right\}$ *and assume*$A_{3}$ *a high assessment*$\left\{\left\{0.5,0.8\right\},\left\{0.2,0.3\right\}\right\}$ *and the other assessment information is the same as Example 3. The method proposed by Zhang et al. [55] and our proposed method are used to solve the Example 4 and the decision results are shown in Table 10.*

From Table 10, it is noted that the ranking order obtained by Zhang et al.’s [55] method changed into $A_{3}>A_{5}>A_{4}>A_{2}>A_{1}$, the best choice changed into $A_{3}$. Besides, the order obtained by our method also be $A_{5}>A_{4}>A_{3}>A_{2}>A_{1}$ and the optimal choice is $A_{5}$, which direct that our method can effectively deal with the extreme values. Hence, our method is more flexible and robust than Zhang et al.’s [55] method.

#### 7.6.2. It Can Determine the Weight Information of Attributes Objectively

In our proposed MADM method, the weight information of attributes is unknown. In other words, our method provides a method to objectively determine weight information of attributes based on the decision matrix. However, the MADM methods in Xu et al. [17] are based on the supposition that the weight vector of attributes is known. In the reality, due to the difficulties of decision-making problems, it is usually difficult to provide attributes’ weights by DMs. When employing our method to choose the optimal alternative, DMs need not provide weight information in advance. Hence, in our decision-making method, the weight vector of attributes is objectively determined by DMs’ original decision matrices, which makes the decision results more reasonable and reliable. Hence, our method is more flexible than Xu et al.’s [17] method.

#### 7.6.3. It Can Consider the Complex Interrelationship among Multiple Attributes

In real decision-making problems, the interrelationship among attributes is usually changeable. To make the final results more reliable, it is necessary to take the interrelationship into consideration when calculating. We propose a multi-attribute decision-making method based on the q-RDHFPWHM operator, which can handle complex interrelationships. To prove this advantage, Example 4 is solved by our method, and the results are shown in Table 11. We can find that the best alternative with different *k* is different. In real decision-making problem, DMs can select a proper *k* according to actual needs.

#### 7.6.4. It Can Effectively Express DM’s Evaluation Comprehensively

The constraint of q-RDHFEs is that of *q*th power of MD and *q*th power of NMD is less than or equal to one. Compared with the DHFs, q-RDHFs can describe larger information space. Basically, q-RDHFs allow DMs to evaluate the alternatives more comprehensively. The example 5 is shown to illustrate the advantage better.

**Example** **5.** *In Example 3, DMs use DHFs to note their assessment. The constraint of DHFSs is the sum of MG and NMG ought to be less than one. Particularly, this constrain cannot be always satisfied. Such that the evaluation value of attribute*$C_{3}$ *of*$A_{2}$ *is changed into*$\left\{\left\{0.8\right\},\left\{0.3,0.8,0.9\right\}\right\}$*. Then, the method proposed by Zhang et al. and us are used to solve Example 5 and the decision results are shown in Table 12.*

As shown in Table 12, the method proposed by Zhang et al. [55] cannot be suitable for Example 5. Our method solves it, and the ranking orders is $A_{5}>A_{3}>A_{4}>A_{2}>A_{1}$. This is because the value $\left\{\left\{0.8\right\},\left\{0.3,0.8,0.9\right\}\right\}$ does not satisfy the constraint of DHFs, as 0.8+0.9=1.7>1. The method proposed by us remains suitable for this example, such as our set q=5, so $0.8^{5}+0.9^{5}=0.9182<1$. Hence, it is effective to deal with DMs’ assessment information by our method.

## 8. Conclusions

This paper proposed a new MADM approach based on q-RDHFs. The main attributes can be summarized into three points. First, to solve the existing methods based on q-RDHFSs only consider the relationship between attribute values, we present a novel MADM method used the proposed q-RDHFPHM operator and q-RDHFPWHM operator, which further consider how to deal with unreasonable or extreme evaluation values of DMs. Second, in most MADM problems, weight vector of attributes is unknown and DMs provide the weight information with difficulty. Based on the entropy measure, we determine the weight information of a set of q-RDHFE, so the above problems can be solved. Finally, a comprehensive novel method to handle MADM problems with q-RDHFPWHM is derived. Meanwhile, a numerical example is given to illustrate how the proposed method can be used to solve assessment of hospitals’ medical quality. Numerical examples and comparative analysis demonstrate how our method is more powerful and feasible than other existing methods.

Compared with existing methods, our proposed method has obvious advantages; however, our method is insufficient to handle decision makers’ interval-valued evaluation information. In addition, we only focus on MADM problems where there are several decision makers. However, as real decision-making problems are becoming more and more complex, more decision makers are necessary for determining the final decision results. Hence, large-scale group decision-making has become a promising research topic [56,57,58]. We will pay more attention to these limitations and strengthen the depth of research. In future works, we will continue our research from three aspects. First, we shall investigate more MADM methods under q-rung dual hesitant fuzzy decision-making environment. Second, in order to handle DMs’ interval-valued information, we shall continue to study interval-valued q-RDHFSs-based MADM method. Third, we shall investigate methods for large-scale group decision-making under q-RDHFSs and interval-valued q-RDHFSs.

## Figures and Tables

**Table 1:** The related studies mentioned.

References	Theory	Characteristics
Fuzzy Sets		
Zadeh [27] (1965)	FSs	The MD is interval [0, 1].
Atanassov [28] (1986)	IFSs	The sum of MD and NMD should be less than or equal to one.
Torra [30] (2010)	HFSs	The MD is denoted by a set of possible values in [0, 1].
Zhu et al. [19] (2012)	DHFSs	The sum of maximum values of MD and NMD is less than or equal to one.
Yager [29] (2014)	PFSs	The square sum of MD and NMD is less than or equal to one.
Yager [18] (2017)	q-ROFSs	The sum of the *q*th power of MD and the *q*th power of NMD does not exceed 1.
Xu et al. [17] (2018)	q-RDHFSs	Both MD and NMD are denoted by multiple values and the sum of *q*th power of maximum MD and *q*th power of maximum NMD does not exceed 1.
Aggregation Operators		
Bonferroni [15] (1950)	BM	It considers the interrelationship among any two arguments.
Sykora [16] (2009)	HEM	It considers the interrelationship among any two arguments.
Yager [37] (2001)	PA	It effectively handles extreme input arguments.
He et al. [38] (2014)	PBM	It takes the advantages of PA and BM.
Peide Liu [39] (2017)	PHEM	It takes the advantages of PA and HEM.
Hara et al. [46] (1998)	HM	It can consider the interrelationship among multiple arguments.
Peide Liu [47] (2019)	PHM	It takes the advantages of PA and HM.

**Table 2:** Medical quality evaluation system.

Index	Implication
Work efficiency (*C*_1_)	Utilization rate of hospital beds
	Average length of hospital stay
	The number of outpatient emergency patients
Medical quality (*C*_2_)	Cure rate, improvement rate and case fatality rate
	Success rate of critically ill rescue
	Satisfaction of nursing service
Workload (*C*_3_)	Number of visits
	Number of hospitalizations

**Table 3:** The q-rung dual hesitant decision matrix.

	*C*_1_	*C*_2_	*C*_3_
*A*_1_	$\left\{\left\{0.3,0.4\right\},\left\{0.6\right\}\right\}$	$\left\{\left\{0.7,0.9\right\},\left\{0.2\right\}\right\}$	$\left\{\left\{0.5,0.6\right\},\left\{0.3\right\}\right\}$
*A*_2_	$\left\{\left\{0.2,0.3\right\},\left\{0.7\right\}\right\}$	$\left\{\left\{0.6,0.7\right\},\left\{0.4\right\}\right\}$	$\left\{\left\{0.7\right\},\left\{0.2,0.3,0.4\right\}\right\}$
*A*_3_	$\left\{\left\{0.5\right\},\left\{0.2,0.3\right\}\right\}$	$\left\{\left\{0.2,0.3,0.4\right\},\left\{0.6\right\}\right\}$	$\left\{\left\{0.5\right\},\left\{0.3,0.4\right\}\right\}$
*A*_4_	$\left\{\left\{0.7,0.8\right\},\left\{0.2\right\}\right\}$	$\left\{\left\{0.6\right\},\left\{0.5\right\}\right\}$	$\left\{\left\{0.5,0.7\right\},\left\{0.1,0.2\right\}\right\}$

**Table 4:** Score values of alternatives $A_{i}\left(i=1,2,3,4\right)$ when $q\in \left[1,5\right]$ based on q-RDHFPWHM operator (*k* = 2).

*q*	$\mathrm{Score}\;\mathrm{Values}\;S\left(d_{i}\right)\left(i=1,2,3\right)$	Ranking Orders
*q* = 1	$S\left(d_{1}\right)=-0.4985$,$\;S\left(d_{2}\right)=-0.5700$, $S\left(d_{3}\right)=-0.5804$,$\;S\left(d_{4}\right)=-0.4127$	$A_{4}>A_{1}>A_{2}>A_{3}$
*q* = 2	$S\left(d_{1}\right)=-0.4424$,$\;S\left(d_{2}\right)=-0.5136$, $S\left(d_{3}\right)=-0.4968$,$\;S\left(d_{4}\right)=-0.308$4	$A_{4}>A_{1}>A_{3}>A_{2}$
*q* = 3	$S\left(d_{1}\right)=-0.4041$,$\;S\left(d_{2}\right)=-0.4334$, $S\left(d_{3}\right)=-0.4001$,$\;S\left(d_{4}\right)=-0.2350$	$A_{4}>A_{3}>A_{1}>A_{2}$
*q* = 4	$S\left(d_{1}\right)=-0.3969$,$\;S\left(d_{2}\right)=-0.3590$, $S\left(d_{3}\right)=-0.3190$,$\;S\left(d_{4}\right)=-0.1910$	$A_{4}>A_{3}>A_{2}>A_{1}$
*q* = 5	$S\left(d_{1}\right)=-0.4140$,$\;S\left(d_{2}\right)=-0.2930$, $S\left(d_{3}\right)=-0.2573$,$\;S\left(d_{4}\right)=-0.1663$	$A_{4}>A_{3}>A_{2}>A_{1}$

**Table 5:** Score values and ranking results with different values of *k* in the q-RDHFPWHM operator (*q* = 3).

*k*	$\mathrm{Score}\;\mathrm{Values}\;S\left(d_{i}\right)\left(i=1,2,3,4\right)$	Ranking Orders
*k* = 1	$S\left(d_{1}\right)=-0.1117$,$\;S\left(d_{2}\right)=-0.3535$, $S\left(d_{3}\right)=-0.3806$,$\;S\left(d_{4}\right)=-0.0675$	$A_{4}>A_{1}>A_{2}>A_{3}$
*k* = 2	$S\left(d_{1}\right)=-0.4041$,$\;S\left(d_{2}\right)=-0.4334$, $S\left(d_{3}\right)=-0.4001$,$\;S\left(d_{4}\right)=-0.2350$	$A_{4}>A_{3}>A_{1}>A_{2}$
*k* = 3	$S\left(d_{1}\right)=-0.4542$,$\;S\left(d_{2}\right)=-0.4636$, $S\left(d_{3}\right)=-0.4060$,$\;S\left(d_{4}\right)=-0.3594$	$A_{4}>A_{3}>A_{1}>A_{2}$

**Table 6:** The decision-making results by different methods.

Methods	$\mathrm{Score}\;\mathrm{Values}\;S\left(d_{i}\right)\left(i=1,2,3,4\right)$	Ranking Orders
Xu et al.’s [17] method based on q-RDHFWHM operator (*t* = 1, *s* = 1, *q* = 3)	$S\left(d_{1}\right)=-0.7206$,$\;S\left(d_{2}\right)=-0.6748$, $S\left(d_{3}\right)=-0.2525$,$\;S\left(d_{4}\right)=0.1998$	$A_{4}>A_{3}>A_{2}>A_{1}$
Our method based on q-RDHFPWHM (*k* = 1, *q* = 3)	$S\left(d_{1}\right)=-0.1117$,$\;S\left(d_{2}\right)=-0.3535$, $S\left(d_{3}\right)=-0.3806$,$\;S\left(d_{4}\right)=-0.0675$	$A_{4}>A_{1}>A_{2}>A_{3}$

**Table 7:** The decision-making results by different methods.

Methods	$\mathrm{Score}\;\mathrm{Values}\;S\left(d_{i}\right)\left(i=1,2,3,4\right)$	Ranking Orders
Wei et al.’s [54] method based on DHPFHWA operator	$S\left(d_{1}\right)=0.7153$,$\;S\left(d_{2}\right)=0.5778$, $S\left(d_{3}\right)=0.5143$,$\;S\left(d_{4}\right)=0.7229$	$A_{4}>A_{1}>A_{2}>A_{3}$
Our method based on q-RDHFPWHM (*k* = 3, *q* = 2)	$S\left(d_{1}\right)=-0.4924$,$\;S\left(d_{2}\right)=-0.5403$, $S\left(d_{3}\right)=-0.5039$,$\;S\left(d_{4}\right)=-0.4237$	$A_{4}>A_{1}>A_{3}>A_{2}$

**Table 8:** The normalized q-rung dual hesitant decision matrix of Example 3.

	*C*_1_	*C*_2_	*C*_3_	*C*_4_
*A*_1_	$\left\{\left\{0.3,0.4\right\},\left\{0.6\right\}\right\}$	$\left\{\left\{0.4,0.5\right\},\left\{0.3,0.4\right\}\right\}$	$\left\{\left\{0.2,0.3\right\},\left\{0.7\right\}\right\}$	$\left\{\left\{0.4,0.5\right\},\left\{0.5\right\}\right\}$
*A*_2_	$\left\{\left\{0.6\right\},\left\{0.4\right\}\right\}$	$\left\{\left\{0.2,0.4,0.5\right\},\left\{0.4\right\}\right\}$	$\left\{\left\{0.2\right\},\left\{0.6,0.7,0.8\right\}\right\}$	$\left\{\left\{0.5\right\},\left\{0.4,0.5\right\}\right\}$
*A*_3_	$\left\{\left\{0.5,0.7\right\},\left\{0.2\right\}\right\}$	$\left\{\left\{0.2\right\},\left\{0.7,0.8\right\}\right\}$	$\left\{\left\{0.2,0.3,0.4\right\},\left\{0.6\right\}\right\}$	$\left\{\left\{0.5,0.6,0.7\right\},\left\{0.3\right\}\right\}$
*A*_4_	$\left\{\left\{0.7\right\},\left\{0.3\right\}\right\}$	$\left\{\left\{0.6,0.7,0.8\right\},\left\{0.2\right\}\right\}$	$\left\{\left\{0.1,0.2\right\},\left\{0.3\right\}\right\}$	$\left\{\left\{0.1\right\},\left\{0.6,0.7,0.8\right\}\right\}$
*A*_5_	$\left\{\left\{0.6,0.7\right\},\left\{0.2\right\}\right\}$	$\left\{\left\{0.2,0.3,0.4\right\},\left\{0.5\right\}\right\}$	$\left\{\left\{0.4,0.5\right\},\left\{0.2\right\}\right\}$	$\left\{\left\{0.2,0.3,0.4\right\},\left\{0.5\right\}\right\}$

**Table 9:** The decision-making results of example 3 by different methods.

Methods	$\mathrm{Score}\;\mathrm{Values}\;S\left(d_{i}\right)\left(i=1,2,3,4,5\right)$	Ranking Orders
Zhang’s [55] method based on DHFMSM operator	$S\left(d_{1}\right)=-0.2046$,$\;S\left(d_{2}\right)=-0.1190$, $S\left(d_{3}\right)=-0.0360$,$S\left(d_{4}\right)=-0.0445$, $S\left(d_{5}\right)=0.0528$	$A_{5}>A_{3}>A_{4}>A_{2}>A_{1}$
Our method based on q-RDHFPWHM (*k* = 3, *q* = 2)	$S\left(d_{1}\right)=-0.7812$,$\;S\left(d_{2}\right)=-0.7545$, $S\left(d_{3}\right)=-0.6648$,$\;S\left(d_{4}\right)=-0.6700$, $S\left(d_{5}\right)=-0.5805$	$A_{5}>A_{3}>A_{4}>A_{2}>A_{1}$

**Table 10:** The decision-making results of example 4 by different methods.

Methods	$\mathrm{Score}\;\mathrm{Values}\;S\left(d_{i}\right)\left(i=1,2,3,4,5\right)$	Ranking Orders
Zhang et al.’s [55] method based on DHFMSM operator (*k* = 2)	$S\left(d_{1}\right)=-0.7584$,$\;S\left(d_{2}\right)=-0.7235$, $S\left(d_{3}\right)=-0.6120$,$\;S\left(d_{4}\right)=-0.6773$, $S\left(d_{5}\right)=-0.6134$	$A_{3}>A_{5}>A_{4}>A_{2}>A_{1}$
Our method based on q-RDHFPWHM (*k* = 2, *q* = 3)	$S\left(d_{1}\right)=-0.7186$,$\;S\left(d_{2}\right)=-0.6869$, $S\left(d_{3}\right)=-0.6056$,$\;S\left(d_{4}\right)=-0.5511$, $S\left(d_{5}\right)=-0.4629$	$A_{5}>A_{4}>A_{3}>A_{2}>A_{1}$

**Table 11:** Score values and ranking results with different values of *k* in the q-RDHFPWHM operator (*q* = 3).

*k*	$\mathrm{Score}\;\mathrm{Values}\;S\left(d_{i}\right)\left(i=1,2,3,4,5\right)$	Ranking Orders
*k* = 1	$S\left(d_{1}\right)=-0.6877$,$\;S\left(d_{2}\right)=-0.6257$, $S\left(d_{3}\right)=-0.5172$,$\;S\left(d_{4}\right)=-0.3811$, $S\left(d_{5}\right)=-0.3358$	$A_{5}>A_{4}>A_{3}>A_{2}>A_{1}$
*k* = 2	$S\left(d_{1}\right)=-0.7186$,$\;S\left(d_{2}\right)=-0.6869$, $S\left(d_{3}\right)=-0.5690$,$\;S\left(d_{4}\right)=-0.5511$, $S\left(d_{5}\right)=-0.4804$	$A_{5}>A_{4}>A_{3}>A_{2}>A_{1}$
*k* = 3	$S\left(d_{1}\right)=-0.7309$,$\;S\left(d_{2}\right)=-0.7074$, $S\left(d_{3}\right)=-0.5865$,$\;S\left(d_{4}\right)=-0.6317$, $S\left(d_{5}\right)=-0.5088$	$A_{5}>A_{3}>A_{4}>A_{2}>A_{1}$
*k* = 4	$S\left(d_{1}\right)=-0.3048$,$\;S\left(d_{2}\right)=-0.2656$, $S\left(d_{3}\right)=0.0456$,$\;S\left(d_{4}\right)=-0.3127$, $S\left(d_{5}\right)=-0.1202$	$A_{3}>A_{5}>A_{2}>A_{1}>A_{4}$

**Table 12:** The decision results of Example 3 by different methods.

Method	$\mathrm{Score}\;\mathrm{Values}\;S\left(d_{i}\right)\left(i=1,2,3,4,5\right)$	Ranking Orders
Zhang’s [55] method based on DHFMSM operator	Cannot be calculated	——
Our method based on q-RDHFPWHM(*k* = 2; *q* = 5)	$S\left(d_{1}\right)=-0.6368$,$\;\left(d_{2}\right)=-0.6167$, $S\left(d_{3}\right)=-0.4454$, $S\left(d_{4}\right)=-0.4497$, $S\left(d_{5}\right)=-0.4233$	$A_{5}>A_{3}>A_{4}>A_{2}>A_{1}$

## Data Availability

Not applicable.

## Appendix A. The Proof Process of Theory 1 in Section 3.1

**Proof.** 

$$n\delta_{i_{j}}d_{i_{j}}=\cup_{\gamma_{i_{j}}\in h_{i_{j}},\eta_{i_{j}}\in g_{i_{j}}}\left\{\left\{{\left(1-{\left(1-\gamma_{i_{j}}^{q}\right)}^{n\delta_{i_{j}}}\right)}^{\frac{1}{q}}\right\},\left\{\eta_{i_{j}}^{n\delta_{i_{j}}}\right\}\right\},$$

and

$$\overset{k}{\underset{j=1}{⊗}}\left(n\delta_{i_{j}}d_{i_{j}}\right)=\cup_{\gamma_{i_{j}}\in h_{i_{j}},\eta_{i_{j}}\in g_{i_{j}}}\left\{\left\{\prod_{j=1}^{k}{\left(1-{\left(1-\gamma_{i_{j}}^{q}\right)}^{n\delta_{i_{j}}}\right)}^{\frac{1}{q}}\right\},\left\{{\left(1-\prod_{j=1}^{k}\left(1-\eta_{i_{j}}^{nq\delta_{i_{j}}}\right)\right)}^{\frac{1}{q}}\right\}\right\}.$$

Therefore

$${\left(\overset{k}{\underset{j=1}{⊗}}\left(n\delta_{i_{j}}d_{i_{j}}\right)\right)}^{1/k}=\cup_{\gamma_{i_{j}}\in h_{i_{j}},\eta_{i_{j}}\in g_{i_{j}}}\left\{\left\{\prod_{j=1}^{k}{\left(1-{\left(1-\gamma_{i_{j}}^{q}\right)}^{n\delta_{i_{j}}}\right)}^{\frac{1}{kq}}\right\},\left\{{\left(1-{\prod_{j=1}^{k}\left(1-\eta_{i_{j}}^{nq\delta_{i_{j}}}\right)}^{\frac{1}{k}}\right)}^{\frac{1}{q}}\right\}\right\}.$$

Further,

$$\underset{1\leq i_{1}<\ldots <i_{k}\leq n}{⊕}{\left(\overset{k}{\underset{j=1}{⊗}}\left(n\delta_{i_{j}}d_{i_{j}}\right)\right)}^{1/k}=\cup_{\gamma_{i_{j}}\in h_{i_{j}},\eta_{i_{j}}\in g_{i_{j}}}$$

$$\left\{\left\{{\left(1-\prod_{1\leq i_{1}<\ldots <i_{k}\leq n}\left(1-\prod_{j=1}^{k}{\left(1-{\left(1-\gamma_{i_{j}}^{q}\right)}^{n\delta_{i_{j}}}\right)}^{\frac{1}{k}}\right)\right)}^{\frac{1}{q}}\right\},\left\{\prod_{1\leq i_{1}<\ldots <i_{k}\leq n}{\left(1-{\prod_{j=1}^{k}\left(1-\eta_{i_{j}}^{nq\delta_{i_{j}}}\right)}^{\frac{1}{k}}\right)}^{\frac{1}{q}}\right\}\right\}.$$

Thus

$$\frac{1}{C_{n}^{k}}\left(\underset{1\leq i_{1}<\cdots <i_{k}\leq n}{⊕}{\left(\overset{k}{\underset{j=1}{⊗}}\left(n\delta_{i_{j}}d_{i_{j}}\right)\right)}^{1/k}\right)=\cup_{\gamma_{i_{j}}\in h_{i_{j}},\eta_{i_{j}}\in g_{i_{j}}}$$

$$\left\{\left\{{\left(1-{\prod_{1\leq i_{1}<\ldots <i_{k}\leq n}\left(1-\prod_{j=1}^{k}{\left(1-{\left(1-\gamma_{i_{j}}^{q}\right)}^{n\delta_{i_{j}}}\right)}^{\frac{1}{k}}\right)}^{{}^{\frac{1}{C_{n}^{k}}}}\right)}^{\frac{1}{q}}\right\},\left\{\prod_{1\leq i_{1}<\ldots <i_{k}\leq n}{\left(1-{\prod_{j=1}^{k}\left(1-\eta_{i_{j}}^{nq\delta_{i_{j}}}\right)}^{\frac{1}{k}}\right)}^{\frac{1}{qC_{n}^{k}}}\right\}\right\}.$$

 □

## Appendix B. The Proof Process of Theory 2 in Section 3.1

**Proof.** Since $d_{i}=d=\left(h,g\right)\left(i=1,2,\ldots ,n\right)$, then $Sup\left(d_{i},d_{j}\right)=1$ for i,j=1,2,…,n; i≠j can be get. Thus, $\delta_{i}=1/n\left(i=1,2,\ldots ,n\right)$ holds for all *i*. According to Theorem 1,

$$q-RDHFPHM^{\left(k\right)}\left(d_{1},d_{2},\ldots ,d_{n}\right)=\cup_{\gamma_{i_{j}}\in h_{i_{j}},\eta_{i_{j}}\in g_{i_{j}}}$$

$$\left\{\left\{{\left(1-{\prod_{1\leq i_{1}<\ldots <i_{k}\leq n}\left(1-\prod_{j=1}^{k}{\left(1-{\left(1-\gamma_{i_{j}}^{q}\right)}^{n\delta_{i_{j}}}\right)}^{\frac{1}{k}}\right)}^{{}^{\frac{1}{C_{n}^{k}}}}\right)}^{\frac{1}{q}}\right\},\left\{\prod_{1\leq i_{1}<\ldots <i_{k}\leq n}{\left(1-{\prod_{j=1}^{k}\left(1-\eta_{i_{j}}^{nq\delta_{i_{j}}}\right)}^{\frac{1}{k}}\right)}^{\frac{1}{qC_{n}^{k}}}\right\}\right\}$$

$$=\cup_{\gamma_{i_{j}}\in h_{i_{j}},\eta_{i_{j}}\in g_{i_{j}}}\left\{\left\{{\left(1-{\prod_{1\leq i_{1}<\ldots <i_{k}\leq n}\left(1-\prod_{j=1}^{k}{\left(1-{\left(1-\gamma_{i_{j}}^{q}\right)}^{n\times \frac{1}{n}}\right)}^{\frac{1}{k}}\right)}^{{}^{\frac{1}{C_{n}^{k}}}}\right)}^{\frac{1}{q}}\right\},\left\{\prod_{1\leq i_{1}<\ldots <i_{k}\leq n}{\left(1-{\prod_{j=1}^{k}\left(1-\eta_{i_{j}}^{nq\times \frac{1}{n}}\right)}^{\frac{1}{k}}\right)}^{\frac{1}{qC_{n}^{k}}}\right\}\right\}$$

$$=\cup_{\gamma_{i_{j}}\in h_{i_{j}},\eta_{i_{j}}\in g_{i_{j}}}\left\{\left\{{\left(1-{\prod_{1\leq i_{1}<\ldots <i_{k}\leq n}\left(1-{\left(\gamma_{i_{j}}\right)}^{k\times \frac{q}{k}}\right)}^{{}^{\frac{1}{C_{n}^{k}}}}\right)}^{\frac{1}{q}}\right\},\left\{\prod_{1\leq i_{1}<\ldots <i_{k}\leq n}{\left(1-{\left(1-\eta_{i_{j}}^{q}\right)}^{k\times \frac{1}{k}}\right)}^{\frac{1}{qC_{n}^{k}}}\right\}\right\}$$

$$=\cup_{\gamma_{i_{j}}\in h_{i_{j}},\eta_{i_{j}}\in g_{i_{j}}}\left\{\left\{{\left(1-{\prod_{1\leq i_{1}<\ldots <i_{k}\leq n}\left(1-{\left(\gamma_{i_{j}}\right)}^{q}\right)}^{{}^{\frac{1}{C_{n}^{k}}}}\right)}^{\frac{1}{q}}\right\},\left\{\prod_{1\leq i_{1}<\ldots <i_{k}\leq n}{\left(\eta_{i_{j}}^{q}\right)}^{\frac{1}{qC_{n}^{k}}}\right\}\right\}$$

$$=\cup_{\gamma_{i_{j}}\in h_{i_{j}},\eta_{i_{j}}\in g_{i_{j}}}\left\{\left\{{\left(1-{\left(1-{\left(\gamma_{i_{j}}\right)}^{q}\right)}^{\frac{1}{C_{n}^{k}}\times C_{n}^{k}}\right)}^{\frac{1}{q}}\right\},\left\{{\left(\eta_{i_{j}}^{q}\right)}^{\frac{1}{qC_{n}^{k}}\times C_{n}^{k}}\right\}\right\}=\left(h,g\right)=d$$

 □

## Appendix C. The Proof Process of Theory 3 in Section 3.1

**Proof.** From Definition 7, we can obtain

$$n\delta_{i_{j}}d^{-}\leq n\delta_{i_{j}}d_{i_{j}},$$

and

$$\overset{k}{\underset{j=1}{⊗}}\left(n\delta_{i_{j}}d^{-}\right)\leq \overset{k}{\underset{j=1}{⊗}}\left(n\delta_{i_{j}}d_{i_{j}}\right).$$

Thus,

$${\left(\overset{k}{\underset{j=1}{⊗}}\left(n\delta_{i_{j}}d^{-}\right)\right)}^{1/k}\leq {\left(\overset{k}{\underset{j=1}{⊗}}\left(n\delta_{i_{j}}d_{i_{j}}\right)\right)}^{1/k},$$

Therefore,

$$\underset{1\leq i_{1}<\ldots <i_{k}\leq n}{⊕}{\left(\overset{k}{\underset{j=1}{⊗}}\left(n\delta_{i_{j}}d^{-}\right)\right)}^{1/k}\leq \underset{1\leq i_{1}<\ldots <i_{k}\leq n}{⊕}{\left(\overset{k}{\underset{j=1}{⊗}}\left(n\delta_{i_{j}}d_{i_{j}}\right)\right)}^{1/k}.$$

Finally,

$$\frac{1}{C_{n}^{k}}\left(\underset{1\leq i_{1}<\cdots <i_{k}\leq n}{⊕}{\left(\overset{k}{\underset{j=1}{⊗}}\left(n\delta_{i_{j}}d^{-}\right)\right)}^{1/k}\right)\leq \frac{1}{C_{n}^{k}}\left(\underset{1\leq i_{1}<\cdots <i_{k}\leq n}{⊕}{\left(\overset{k}{\underset{j=1}{⊗}}\left(n\delta_{i_{j}}d_{i_{j}}\right)\right)}^{1/k}\right),$$

which represents $x\leq q-RDHFPHM^{\left(k\right)}\left(d_{1},d_{2},\ldots ,d_{n}\right)$. □

Analogously, $q-RDHFPHM^{\left(k\right)}\left(d_{1},d_{2},\ldots ,d_{n}\right)\leq y$ can be proved. Therefore, Theorem 3 is proved completely.

## Appendix D. The Proof Process of Theory 4 in Section 3.2

**Proof.** 

$$n\sigma_{i_{j}}d_{i_{j}}=\cup_{\gamma_{i_{j}}\in h_{i_{j}},\eta_{i_{j}}\in g_{i_{j}}}\left\{\left\{{\left(1-{\left(1-\gamma_{i_{j}}^{q}\right)}^{n\sigma_{i_{j}}}\right)}^{\frac{1}{q}}\right\},\left\{\eta_{i_{j}}^{n\sigma_{i_{j}}}\right\}\right\}.$$

and

$$\overset{k}{\underset{j=1}{⊗}}\left(n\sigma_{i_{j}}d_{i_{j}}\right)=\cup_{\gamma_{i_{j}}\in h_{i_{j}},\eta_{i_{j}}\in g_{i_{j}}}\left\{\left\{\prod_{j=1}^{k}{\left(1-{\left(1-\gamma_{i_{j}}^{q}\right)}^{n\sigma_{i_{j}}}\right)}^{\frac{1}{q}}\right\},\left\{{\left(1-\prod_{j=1}^{k}\left(1-\eta_{i_{j}}^{nq\sigma_{i_{j}}}\right)\right)}^{\frac{1}{q}}\right\}\right\}.$$

Then,

$${\left(\overset{k}{\underset{j=1}{⊗}}\left(n\sigma_{i_{j}}d_{i_{j}}\right)\right)}^{1/k}=\cup_{\gamma_{i_{j}}\in h_{i_{j}},\eta_{i_{j}}\in g_{i_{j}}}\left\{\left\{\prod_{j=1}^{k}{\left(1-{\left(1-\gamma_{i_{j}}^{q}\right)}^{n\sigma_{i_{j}}}\right)}^{\frac{1}{kq}}\right\},\left\{{\left(1-{\prod_{j=1}^{k}\left(1-\eta_{i_{j}}^{nq\sigma_{i_{j}}}\right)}^{\frac{1}{k}}\right)}^{\frac{1}{q}}\right\}\right\}.$$

Further,

$$\underset{1\leq i_{1}<\cdots <i_{k}\leq n}{⊕}{\left(\overset{k}{\underset{j=1}{⊗}}\left(n\sigma_{i_{j}}d_{i_{j}}\right)\right)}^{1/k}=\cup_{\gamma_{i_{j}}\in h_{i_{j}},\eta_{i_{j}}\in g_{i_{j}}}.$$

$$\left\{\left\{{\left(1-\prod_{1\leq i_{1}<\ldots <i_{k}\leq n}\left(1-\prod_{j=1}^{k}{\left(1-{\left(1-\gamma_{i_{j}}^{q}\right)}^{n\sigma_{i_{j}}}\right)}^{\frac{1}{k}}\right)\right)}^{\frac{1}{q}}\right\},\left\{\prod_{1\leq i_{1}<\ldots <i_{k}\leq n}{\left(1-{\prod_{j=1}^{k}\left(1-\eta_{i_{j}}^{nq\sigma_{i_{j}}}\right)}^{\frac{1}{k}}\right)}^{\frac{1}{q}}\right\}\right\}.$$

Finally,

$$\frac{1}{C_{n}^{k}}\left(\underset{1\leq i_{1}<\cdots <i_{k}\leq n}{⊕}{\left(\overset{k}{\underset{j=1}{⊗}}\left(n\sigma_{i_{j}}d_{i_{j}}\right)\right)}^{1/k}\right)=\cup_{\gamma_{i_{j}}\in h_{i_{j}},\eta_{i_{j}}\in g_{i_{j}}}.$$

$$\left\{\left\{{\left(1-{\prod_{1\leq i_{1}<\ldots <i_{k}\leq n}\left(1-\prod_{j=1}^{k}{\left(1-{\left(1-\gamma_{i_{j}}^{q}\right)}^{n\sigma_{i_{j}}}\right)}^{\frac{1}{k}}\right)}^{{}^{\frac{1}{C_{n}^{k}}}}\right)}^{\frac{1}{q}}\right\},\left\{\prod_{1\leq i_{1}<\ldots <i_{k}\leq n}{\left(1-{\prod_{j=1}^{k}\left(1-\eta_{i_{j}}^{nq\sigma_{i_{j}}}\right)}^{\frac{1}{k}}\right)}^{\frac{1}{qC_{n}^{k}}}\right\}\right\}.$$

 □

## Appendix E. The Proof Process of Theory 5 in Section 3.2

**Proof.** 

$$n\sigma_{i_{j}}d^{-}\leq n\sigma_{i_{j}}d_{i_{j}},$$

and

$$\overset{k}{\underset{j=1}{⊗}}\left(n\sigma_{i_{j}}d^{-}\right)\leq \overset{k}{\underset{j=1}{⊗}}\left(n\sigma_{i_{j}}d_{i_{j}}\right).$$

Thus,

$${\left(\overset{k}{\underset{j=1}{⊗}}\left(n\sigma_{i_{j}}d^{-}\right)\right)}^{1/k}\leq {\left(\overset{k}{\underset{j=1}{⊗}}\left(n\sigma_{i_{j}}d_{i_{j}}\right)\right)}^{1/k}.$$

Therefore,

$$\underset{1\leq i_{1}<\ldots <i_{k}\leq n}{⊕}{\left(\overset{k}{\underset{j=1}{⊗}}\left(n\sigma_{i_{j}}d^{-}\right)\right)}^{1/k}\leq \underset{1\leq i_{1}<\ldots <i_{k}\leq n}{⊕}{\left(\overset{k}{\underset{j=1}{⊗}}\left(n\sigma_{i_{j}}d_{i_{j}}\right)\right)}^{1/k}.$$

Finally,

$$\frac{1}{C_{n}^{k}}\left(\underset{1\leq i_{1}<\cdots <i_{k}\leq n}{⊕}{\left(\overset{k}{\underset{j=1}{⊗}}\left(n\sigma_{i_{j}}d^{-}\right)\right)}^{1/k}\right)\leq \frac{1}{C_{n}^{k}}\left(\underset{1\leq i_{1}<\cdots <i_{k}\leq n}{⊕}{\left(\overset{k}{\underset{j=1}{⊗}}\left(n\sigma_{i_{j}}d_{i_{j}}\right)\right)}^{1/k}\right),$$

which implies that $x\leq q-RDHFPWHM^{\left(k\right)}\left(d_{1},d_{2},\ldots ,d_{n}\right)$. □

In the same way, $q-RDHFPWHM^{\left(k\right)}\left(d_{1},d_{2},\ldots ,d_{n}\right)\leq y$ can be proved. Therefore, Theorem 5 is proved completely.
